# European agricultural pest management practices following the Green Deal – gaps and challenges

**DOI:** 10.3389/finsc.2026.1901258

**Published:** 2026-07-22

**Authors:** Adalbert Balog, Hugh D. Loxdale, Artúr Botond Csorba, Sándor Keszthelyi

**Affiliations:** 1 Department of Horticulture, Faculty of Technical and Human Sciences, Sapientia Hungarian University of Transylvania, Târgu Mureş/Corunca, Romania; 2 Department of Agrobiology, Institute of Biology, Faculty of Sciences, University of Pécs, Pécs, Hungary; 3 School of Biosciences, Cardiff University, Cardiff, United Kingdom; 4 Department of Agronomy, Institute of Agronomy, Hungarian University of Agriculture and Life Sciences, Kaposvár, Hungary

**Keywords:** biocontrol, CAP eco-schemes, European Green Deal, farm to fork, IPM, pesticides, sustainable use directive, sustainable use regulation

## Abstract

The European Green Deal, the main aim of which is to substantially change the agricultural production systems through the Farm to Fork (F2F) Strategy, has caused the most significant reconsideration of pest management in the European Union (EU) in a generation. Since 2020, several regulatory and policy measures have affected pest management decisions, including revised risk assessment procedures for plant protection products, restrictions on certain emergency derogations, updated rules facilitating microbial biological control agents, and the integration of IPM (Integrated Pest Management)-related measures into the Common Agricultural Policy (CAP) eco-schemes. At the same time, implementation has been challenging. The proposed Sustainable Use Regulation (SUR), which aimed to introduce binding pesticide reduction targets and additional restrictions in sensitive areas, was withdrawn in 2024 following political disagreement within the EU Commission and concerns regarding implementation feasibility. As a result, pesticide governance remains primarily based on the Sustainable Use Directive (SUD), national action plans, and CAP-supported measures. This mini review synthesizes the post Green Deal policy timeline, identifies regulatory factors that directly affect farmers’ pest management choices, examines emerging quantitative trends, and discusses practical implications for industry, advisory systems, and growers. Particular attention is given to the opportunities and limitations of Harmonized Risk Indicators (HRIs) – notably their sales-based nature and limited capacity to reflect actual ecological exposure, alongside uneven IPM implementation among Member States and emerging climate-driven pest pressures.

## 1 Introduction

### 1.1 Green Deal ambitions meet plant-health realities

The Farm to Fork Strategy (F2F) placed pest management as a principal objective to be achieved by the EU’s ‘through sustainability transition’, announcing two headline (non-binding at EU level) targets: (i) reducing pesticides to 50% usage at all levels to decrease risk of these chemical to humans, livestock and wildlife; and (ii), the implementation of more biological and IPM (Integrated Pest Management) methods by the year 2030 (1). Progress is tracked using harmonized risk indicators alongside other statistics (2, 3). The pesticide transition is linked with parallel Green Deal objectives of biodiversity protection and a 25% organic-area involvement through creating strong policy signals to substitute or optimize pesticide use while maintaining plant-health security (4, 5).

### 1.2 Policy timeline since 2020: from ambition to contested implementation

2020–2022: Agenda setting and first regulatory upgrades. The Commission’s F2F announcement in 2020 set the direction of travel. In June 2022, the Commission tabled the SUR to replace the SUD with binding EU-level reduction targets and measures such as sensitive-area restrictions and stronger IPM enforcement. In parallel, the EU adopted a package of regulations revising approval criteria and data requirements for the use of microorganisms in plant protection, explicitly in order to speed safe access to biological alternatives (6, 7).

2023: Scientific and judicial tightening. EFSA issued updated guidance for bees, expanding and standardizing the protection goals and methods used in risk assessment of plant protection products (PPP). Separately, the EU Court of Justice ruled that Member States can no longer grant emergency derogations for the placing on the market and use of seeds treated with neonicotinoids banned at EU level, thereby narrowing a frequently used pathway for continued use of certain hazardous compounds (8, 9).

### 1.3 Late 2023–2024: SUR debate and withdrawal

The SUR proposal became increasingly controversial during 2023, with concerns raised by farmers, agricultural organizations, and several Member States regarding potential production risks, economic impacts, and implementation challenges. These debates occurred alongside widespread farmer demonstrations in several EU countries in early 2024, which contributed to broader political pressure regarding agricultural regulation. Following the rejection of the proposal by the European Parliament in late 2023, the European Commission withdrew the SUR initiative in February 2024.

The withdrawal did not remove existing pesticide sustainability obligations. The Sustainable Use Directive (Directive 2009/128/EC) remains applicable, requiring Member States to implement National Action Plans and promote IPM principles. The post-SUR period has therefore shifted attention toward strengthening existing mechanisms, improving implementation monitoring, and using CAP instruments to encourage reduction of pesticide usage (10, 11).

### 1.4 2023–2027: CAP eco-schemes launch and evolve

The reformed CAP (2023–2027) embedded eco-schemes and agricultural environment measures that many Member States use to support IPM practices (e.g., mechanical weeding, precision application, biological control) beyond baseline requirements, with a majority of national Strategic Plans explicitly including pesticide reduction options (12).

The Commission’s harmonized risk indicators (HRIs) are the primary EU-level measures for pesticide transition progress. On 14 August 2025, the Commission reported that HRI-1 (use and risk proxy based on sales weighted by hazard category) declined by 61% versus the 2011–2013 baseline and by ~5% versus 2022; meanwhile, HRI-2 (emergency authorizations) decreased by ~5% relative to the baseline and ~13% year-over-year, amid tighter scrutiny of derogations.

Nevertheless, these indicators should be interpreted with caution because they are largely based on pesticide sales data weighted by hazard categories and do not fully capture real-world exposure, ecological effects, or chronic impacts. Therefore, HRIs provide useful policy signals but should be complemented with additional environmental and agronomic indicators (13).

This brief overview article discusses, importantly, the main achievements, limitations, and unresolved challenges emerging from the Green Deal transition in European pest management, with particular emphasis on regulatory developments, IPM implementation, biological control, and regional differences.

## 2 Regulatory guidelines with direct implications for pest management

The 2022 package (including Commission Regulation (EU) 2022/1438 and linked implementing acts) updated approval criteria and data requirements to better reflect microbial biology, placing microbial usage at the center of risk assessment and in practice streamlining evaluation where appropriate. The Commission explicitly framed these changes as a way to “facilitate the authorization” of biological PPPs and diversify farmers’ options to replace chemical pesticides. Member States and EU agencies are now implementing these rules, and more than 60 microbial active substances have been approved within the EU (14). The next important challenges include the pollinator protection perspective directly linked with Green Deal regulations. EFSA’s 2023 updated new directive about how chronic, sub-lethal and colony-level effects on pollinator species are addressed in PPP assessment for honey-, bumble- and solitary bees. For registrants, this means new data packages and potentially tighter mitigation measures; for farmers, it can translate into label changes (e.g., time-of-day restrictions and buffer zones) and a shift toward less risky (i.e. collaterally-toxic) products or non-chemical methods where feasible (15). In the January 2023 CJEU ruling, the scope for repeated national emergency authorizations was cut, especially for banned neonicotinoid seed treatments, creating stronger motivations to adopt IPM and alternative controls for pests such as aphids infesting sugar beet. Over time, this reduces Member State discretion to bridge gaps with derogations and increases pressure on innovation, advisory services, and resistant crop varieties (8). SUR’s withdrawal has prevented the introduction of binding EU-level reduction targets and sensitive-area pesticide-free zones. Yet IPM remains mandatory under the SUD, and enforcement can be strengthened via conditionality and controls; consequently, practical pressure now shifts to national action plans and CAP levers rather than a single EU regulation (8).

## 3 IPM mainstreaming through CAP eco-schemes and advisory systems

With CAP 2023–2027, most Member States embedded eco-schemes or agricultural environment measures that explicitly support pesticide reduction e.g., mechanical/thermal weeding, diverse rotations, flowering strips, trap crops, in-season monitoring/threshold-based decisions, precision application technologies, and adoption of biocontrol measures. It was also shown that while the adoption of IPM principles is obligatory in the EU, their implementation has limitations given the considerable regulations variation among EU Member States and the limited development of crop-specific guidelines (16). The adoption of IPM strategies within the EU is also problematic because of several internal challenges (i.e. limited research funds in pest control and limited expertise, knowledge transfer at all levels, and networking (17) and external issues (climate change, development of pesticide resistance) (8, 9, 18) (**Table 1**).

**Table 1:** Examples of IPM implementation approaches in selected EU member states.

Country	Pesticide use trend	Main IPM developments	Examples of innovation	Main challenges
Denmark	Strong decline	Strong pesticide risk reduction programmes	Taxation, digital records, decision-support systems	Economic pressure on smaller farms
The Netherlands	Stable/low use	Highly developed greenhouse IPM	Biological control, automation, precision monitoring	High technology costs and export requirements
France	Moderate decline	Expansion of biocontrol and reduced pesticide use	Biological products, alternative management systems	Adoption barriers and efficacy concerns
Spain	Mixed	Advanced IPM in protected crops	Greenhouse monitoring and biological control	Uneven implementation between sectors
Germany	Moderate decline	Research-based IPM approaches	Ecological management and precision technologies	Farmer acceptance and regulatory complexity

Trends are based on national pesticide sales data and risk indicators as reported in respective Member State National Action Plans; time periods and measurement methods vary between countries.

*Achievements:*

- Sharp reductions in pesticide residues in exported produce.
- Models of climate-neutral and low-input farming systems.

Examples from different Member States illustrate these differences:

- Denmark has implemented strong pesticide reduction policies supported by taxation mechanisms, advisory systems, and digital reporting tools. These measures have contributed to lower pesticide risk indicators but also created economic challenges for some producers.
- The Netherlands has developed advanced greenhouse IPM systems integrating biological control agents such as hymenopterous parasitoid wasps, climate-controlled production, monitoring technologies, and precision management. These systems demonstrate the potential of technology-intensive IPM but require substantial investment.
- France has promoted biocontrol development and pesticide reduction programmes, although adoption remains influenced by concerns regarding efficacy, labour requirements, and economic feasibility.
- Spain shows substantial variation between production systems. Greenhouse horticulture has achieved advanced IPM adoption, whereas intensive open-field production faces greater challenges due to pest pressure and climatic variability.
- Germany has invested in ecological pest management research and IPM development, although implementation remains influenced by farmer acceptance and regulatory uncertainty.

Regulatory signals (i.e. management guidance related to bees, emergency-use constraints, microbial organism facilitation) are accelerating portfolio diversification: hence, more microbial and other biological methods are now seen to be entering the market, increasing focus on lower risk activities, whilst meanwhile placing greater reliance on cultural and mechanical controls. The Commission’s own framing of the 2022 microorganism package emphasized the intent to “provide EU farmers with additional options to replace chemical plant protection products” (14).

Although aerial spraying remains restricted in the EU, precision technologies involving sensor-guided sprayers, variable rate application, and data-driven decision support are being developed through CAP funding streams and eco-schemes to reduce off-target losses and improve timing. Such approaches operationalize IPM strategies at the larger scale, following the principle of “use only as needed, [and] as precisely as possible”. Agriculture and rural development under the SUD means that professional users must keep records, receive training, and have equipment inspected; thus, Member States are increasingly aligning farm advisory services with IPM diagnostics and economic thresholds, with CAP funds supporting advisory capacity (10).

## 4 What the indicators tell us and what they don’t

The August 2025 HRI update shows continued declines: HRI-1 down ~61% vs. 2011–2013 and HRI-2 down ~5% over the same period, with a ~13% year-over-year drop in 2023 emergency authorizations. These reductions can reflect product mix shifts (e.g., away from “more hazardous” categories) as well as absolute use changes. Country-level variability remains large and depends on crop mix, pest pressure (including climate-change-driven outbreaks), and national CAP design choices.

In this light, a reduction in HRI-1 should not be interpreted as equivalent to a proportional reduction in terms of ecological damage and risk. Some scientific and stakeholder discussions have highlighted that sales-based indicators may oversimplify complex exposure pathways and may not fully represent differences between synthetic and natural substances. A balanced evaluation should combine HRIs with environmental monitoring, resistance development data, biodiversity indicators, and crop productivity assessments (13).

Case study threads through examples of ongoing transitions:

- Sugar beet aphid control post-neonicotinoids: With emergency seed treatment derogations shortened, sugar sectors are doubling down on tolerant plant varieties, monitoring, and non-systemic/in-season controls, spotlighting the need for rapid authorization of alternatives and improved forecasting (8).
- Pollinator-sensitive horticulture: Revised bee guidance has prompted tighter risk mitigation on labels and stimulated adoption of physical exclusion (nets), timing measures (safety applications), and selective biocontrol in orchards and greenhouses (15).
- Organic and low-input systems: F2F’s 25% organic target indirectly increases demand for biocontrol and resistant crop varieties and for advisory systems that manage pests primarily through prevention and ecosystem services (4).

## 5 Strategic implications and open issues

- Regulatory pathway for the ‘next SUR’. The Commission’s withdrawal of the 2022 SUR does not obliterate the 2030 reduction ambitions; a re-scoped initiative is expected, and Member States’ uneven progress keeps political pressure high. Stakeholders must expect renewed proposals combining binding elements with stronger support for alternatives to continued widespread pesticide usage and data systems.
- Scale-up of biological pest control beyond microorganisms. While microbial agents used in pest control have a clearer pathway, additional fast track frameworks for other biological categories (e.g., semiochemicals, natural substances, RNA-based products) remain under discussion, with EU Parliamentary signals supportive of acceleration of such approaches (8, 19, 20).
- From IPM obligation to IPM verification. Deeper integration of digital logs, monitoring evidence, and threshold-based decision documentation are expected, potentially linked to eco-scheme eligibility and conditionality checks under the CAP (21, 22).
- Risk assessment evolution. Implementation of the 2023 bee guidance will continue to reshape product availability and mitigation measures and further pollinator and non-target organism guidance updates may follow as evidence and policy evolve (15).

## 6 Central and Eastern Europe: rising pest pressure and adaptive gaps

Central and Eastern European (CEE) countries face particular challenges in implementing sustainable pest management strategies. Countries such as Poland, Hungary, Romania, Bulgaria, Slovakia, and the Baltic States experience increasing pest pressures associated with warmer temperatures, longer growing seasons, and changing pest distributions. Common challenges include:

- Less developed IPM infrastructure.
- Lower investment in precision farming.
- Fragmented advisory services and farmer education.
- Reliance on generic chemical control methods.

Climate-driven pest trends in Central and Eastern European countries are presented in **Table 2**.

**Table 2:** Climate-driven pest trends in Central and Eastern European countries.

Country	Important climate-influenced pest issues	Observed trends
Poland	Aphids, Colorado potato beetle, *Drosophila suzukii*	Earlier emergence and increased virus risks
Hungary	Western corn rootworm, spider mites	Expansion associated with drought and heat stress
Romania	*Tuta absoluta*, codling moth, maize pests	Increased generation numbers under warmer conditions
Bulgaria	Fruit flies and tomato pests	Northward expansion of Mediterranean pests
Lithuania & Latvia	Forest pests and aphid-transmitted viruses	Longer vegetation periods increasing pressure

While the above examples illustrate progress in several Western European countries, the situation differs considerably in Central and Eastern European (CEE) Member States, where lower investment in IPM infrastructure, fragmented advisory services, and more limited research capacity compound the challenges of climate-driven pest pressures.

These trends demonstrate why climate adaptation aims should be integrated with the development of IPM approaches. Without improved monitoring, forecasting, and advisory support, climate-driven pest expansion may increase reliance on chemical control.

## 7 Conclusions

The EU’s goals in relation to Green Deal regulations were primarily focused on reducing the environmental and climate footprint of the EU countries over the short term, thereby ensuring food system security and resilience in the face of climate change, maintenance of biodiversity, in turn leading to a global transition towards competitive sustainability from farm to fork and tapping into new research and practical pest control opportunities (2, 23). Since the implementation of the Green Deal, European pest management has moved along three reinforcing tracks: (i) regulatory tightening where risks are high (neonicotinoid derogations; pollinator protection), (ii) regulatory facilitation for safer alternatives (microbial biologicals), and (iii) economic incentives and advice to mainstream IPM approaches (CAP eco-schemes). The failure of the SUR delayed binding EU-level targets, but did not halt the transition: the SUD still mandates IPM, HRIs show progress, and Member States have the political tools and pressure to keep moving forward. However, the unequal funding applications, and restrictions in research and agricultural development will have negative effects on implementation. Clearly, the next five years will ultimately be defined by how fast biological pest control agents (i.e. entomopathogenic fungi, hymenopterous parasitoid wasps, arthropod predators) are employed above and beyond the use of microorganisms, as well as how credibly IPM is verified and rewarded, and whether a reshaped legislative package can lock in durable reductions without compromising plant health resilience (10).
